# A Halt Called

**Published:** 1897-08

**Authors:** 


A Halt Called.--Texas towns seem to have gone daft on the subject of medical colleges. Privileges easily acquired are apt to be abused; and so long as the State will grant a charter for a medical college (or for almost anything else, provided, always, that the small sum of $10 is forthcoming) upon the application of any two or three persons, we need not be surprised to see medical colleges springing up at every town. Any two or thiee doctors can organize themselves into a "Faculty," and for $10 secure the State's consent to call themselves a medical college. There are in most towns certain doctors who are ambitious to be called "Professor"; there is fascination in the name;—it is a fashionable mode of advertising; they know so much that they are impatient to impart a portion of their wisdom to others; to any who will take it—and pay for it, and hence the multiplication of the evil, for it is a great evil, goes steadily on.

The last town in Texas to develop symptoms of the mania is Dallas. For months there have been rumors of a coming college at Dallas. We had learned some months age that Drs. Milliken and Rosser contemplated establishing a small school at Dallas for post-graduate instruction, but we were not aware that their ambition extended to a full-fledged medical college. In fact, the first intimation we had of such a scheme came recently, in the shape of a circular letter, from a large number of Dallas physicians, earnestly protesting against it, as something calculated to bring the profession into disrepute. The protest is very earnest and energetic. and it expresses our sentiments to the letter. The great evil of the day is—easy matriculation. easy graduation—cheap doctors. The effect is inevitable. The very title "doctor" will become a by-word and a reproach.

We reproduce the circular in this issue, and endorse it fully.

We also reproduce the burlesque announcement of a college at Weatherford. Ridicule is a powerful weapon. In this instance it is to be hoped that the college spirit will be laid, never to show its head again in Texas.

But, as there are said to be two sides to every question, there are two sides to the Dallas college enterprise. If Drs. Milliken and Rosser—both of whom are accomplished physicians and surgeons—and who have had, and profited by the benefit of special teaching in New York and elsewhere—think they can do any good, and incidentally promote their own interests by taking a limited number of country physicians as students for a brief season of clinical instruction,—as we had understood was their intention, we see no impropriety in their doing so. If they see that they have facilities for instructing physicians in methods of diagnosis and in the *technique* of the more advanced operations—something that is understood by very few of the doctors in the smaller towns,—and advertise to that effect, and go ahead and have class after class under instruction in their hospitals or infirmaries (we learn that they have access to or control of several institutions), the course in our estimation is not only not censurable, but is praiseworthy, and will doubtless result in much good. It must be remembered that every country doctor cannot leave home and attend a course of post graduate instruction in New York or Philadelphia or St. Louis, and yet, if they are not posted on certain details—their only alternative is to send a large number of their patients to some city specialist. It is not a bad idea. Facilities for acquiring this special knowledge—offered by competent teachers—near home and on reasonable terms cannot fail to attract considerable patronage, chiefly from young doctors and from older ones living remote from medical centers.

Dr. Armstrong, in *Texas Practitioner*, inveighs heavily against the enterprise of Drs. Milliken and Rosser, and says—"The scheme was conceived in vice and brought forth in iniquity. Among the original promoters there is not a man who is now or ever was entitled to any recognition." We beg to challenge this remark. If, as we understand is the case, Drs. Rosser & Milliken are referred to as the promoters of this scheme, the remark is gratuitous and not true. Both the gentlemen named above are worthy of and have received most distinguished recognition by the State Medical Association. They are educated gentlemen in excellent standing, professionally and socially, and not unknown to fame. They are both Texas men, and Texas has no reason to be ashamed of them as her sons. Dr. Rosser is too well known to our readers to need an introduction; nevertheless, in face of Dr. Armstrong's attack it is due Dr. Rosser to state for the information of those who do not know the fact, that he received "recognition" to the extent of being elected first vice-president of the State Medical Association; and has been superintendent of one of the State Lunatic Asylums. He delivered the valedictory to the graduating class of his Alma Mater, Louisville Medical College, last year, and by invitation delivered a course of lectures on insanity before the Gross Medical College; but as Dr. Milliken has only recently returned to Texas, after an absence of several years, spent in New York, where, from a student in the Polyclinic, he became a teacher—we want to recall to the memory of our readers and of Dr. Armstrong especially, the very cordial "recognition" given Dr. Milliken by the Texas State Medical Association, at Tyler, Texas, in April, 1890.

See Transactions of the State Medical Association, 1890.

"On motion of Dr. E. P. Becton, Dr. Samuel E. Milliken, of New York, was invited to a seat in the Convention, and given the privileges of membership. He exhibited some colored drawings illustrating Bassini's method of operating for the radical cure of hernia. On motion a vote of thanks was tendered Dr. Milliken," and by the Medical Council he was made an honorary member.

The above would indicate that a few years ago the profession of Texas thought Dr. Milliken "worthy of recognition," and we have not heard of his having disgraced himself or done anything to forfeit the good opinion of his Texan colleages. We do not hesitate to say we believe both Drs. Milliken and Rosser would make good teachers of medicine, and would, moreover, wear becomingly the honors of a professorship; but they should go to a larger town than Dallas—to some already "medicine center" and forego their ambition to convert Dallas into a rival of New York, New Orleans, St. Louis or Philadelphia. Dr. Armstrong's "conceived in vice and brought forth in iniquity," is unjust and savors somewhat of prejudice, or—shall we say—envy?

The circular letter referred to, is timely and to the point. A halt has been called, and we hope the call will be heeded. We agree with the signers that if seventy-five per cent. of the colleges already existing, and one hundred per cent. of those that are conducted for what can be made out of them were abolished, it would be better for the profession and far better for the public, and infinitely better for the scores of misguided, misled young men who never ought to attempt the study of medicine, but should stick to the plow.

If our information has been correct, that is, that Drs. Milliken and Rosser contemplated only taking small classes for private instruction in their practice, it would look as if mountains had been made of mole hills. But we confess we are not posted as to the extent of the enterprise contemplated.

