# Erratum

**Published:** 1897-09

**Authors:** 


Erratum.—In our last issue, reference was made to the meeting of the State Medical Association at Tyler in 1890, and to the Transactions of 1890. It should have been 1892, in both instances.

---

With the fate of Professors West and Clopton before them, the uncertain tenure of office, the small salary, it may be asked, can the better element of the Texas medical profession be induced to accept the chairs made vacant by their summary dismissal?

